# Genetically Encoded Self-Assembling Iron Oxide Nanoparticles as a Possible Platform for Cancer-Cell Tracking

**DOI:** 10.3390/pharmaceutics13030397

**Published:** 2021-03-16

**Authors:** Maria V. Efremova, Silviu-Vasile Bodea, Felix Sigmund, Alevtina Semkina, Gil G. Westmeyer, Maxim A. Abakumov

**Affiliations:** 1 Department of Chemistry & TUM School of Medicine, Technical University of Munich (TUM), 81675 Munich, Germany; silviu@westmeyerlab.org (S.-V.B.); felix.sigmund@helmholtz-muenchen.de (F.S.); gil.westmeyer@tum.de (G.G.W.); 2 Institute for Synthetic Biomedicine, Helmholtz Center Munich, 85764 Neuherberg, Germany; 3 Department of Medical Nanobiotechnology, Pirogov Russian National Research Medical University, 117997 Moscow, Russia; alevtina.semkina@gmail.com; 4 V.P. Serbskiy National Medical Research Center of Psychiatry and Narcology, 119034 Moscow, Russia; 5 Laboratory “Biomedical Nanomaterials”, National University of Science and Technology “MISiS”, 119049 Moscow, Russia

**Keywords:** genetically controlled imaging reporters, biogenic iron oxide nanoparticles, visualization of cancer cells, encapsulins, magnetic resonance imaging, fluorescence, cell tracking

## Abstract

The study of growth and possible metastasis in animal models of tumors would benefit from reliable cell labels for noninvasive whole-organism imaging techniques such as magnetic resonance imaging. Genetically encoded cell-tracking reporters have the advantage that they are contrast-selective for viable cells with intact protein expression machinery. Besides, these reporters do not suffer from dilution during cell division. Encapsulins, which are bacterial protein nanocompartments, can serve as genetically controlled labels for multimodal detection of cells. Such nanocompartments can host various guest molecules inside their lumen. These include, for example, fluorescent proteins or enzymes with ferroxidase activity leading to biomineralization of iron oxide inside the encapsulin nanoshell. The aim of this work was to implement heterologous expression of encapsulin systems from *Quasibacillus thermotolerans* using the fluorescent reporter protein mScarlet-I and ferroxidase IMEF in the human hepatocellular carcinoma cell line HepG2. The successful expression of self-assembled encapsulin nanocompartments with functional cargo proteins was confirmed by fluorescence microscopy and transmission electron microscopy. Also, coexpression of encapsulin nanoshells, ferroxidase cargo, and iron transporter led to an increase in T_2_-weighted contrast in magnetic resonance imaging of HepG2 cells. The results demonstrate that the encapsulin cargo system from *Q. thermotolerans* may be suitable for multimodal imaging of cancer cells and could contribute to further in vitro and in vivo studies.

## 1. Introduction

Many advances in cancer treatment would come from a better understanding of tumor biology, particularly the elucidation of carcinogenesis mechanisms in preclinical studies. Highly sensitive molecular imaging of living cells could provide the means to study the formation and growth of metastases in a whole-body context in animal models [1,2,3]. The orthotopic transplantation approach is widely used to simulate, for example, cancer invasion and metastases when cancer cells interact with stromal components, including extracellular matrices, endothelial cells, fibroblasts, and various types of immune cells [4]. Currently, the primary method of live-cell imaging is direct labeling of cells with a probe or contrast agent before transplantation [5,6]. Quantum dots and fluorophores can be used for optical monitoring [7,8,9]. Radionuclides are used in positron emission tomography [10,11], and superparamagnetic iron oxide nanoparticles (SPION) are used in magnetic resonance imaging (MRI) [12,13]. However, any synthetic contrast agent for cell labeling has a critical drawback—it dilutes as the cells divide, which leads to loss of the signal after several cycles of divisions. In contrast, genetically encoded reporters propagate to daughter cells with each cell division. Moreover, because genetically encoded reporters rely on essential cellular processes, their signal is selective for viable cells [14,15].

The most commonly studied genetically encoded labels use an optical signal generated by either bioluminescent (e.g., firefly luciferase) or fluorescent (e.g., jellyfish green fluorescent protein (GFP)) reporter proteins [16]. Although these methods have very high sensitivity [17,18], light is highly scattered in biological tissues, limiting the use of these methods to a depth of about a millimeter [19]. The use of the near-infrared fluorescent proteins can improve tissue penetration depth only to some extent [20,21]. Thus, genetically encoded bioluminescent or fluorescent labels are mainly suitable for reanalysis of tumors ex vivo in combination with histological methods [22].

MRI has the advantage of deep tissue penetration with relatively high (up to 100 µm) spatial resolution [23]. As for genetically encoded reporters for MRI [24], various metalloproteins, such as methemoglobin [25], transferrin [26], cytochrome P450-BM3 [23], and ferritin [27], are overexpressed for T_2_-weighted contrast generation. Ferritin, which was the first protein to induce MRI contrast in the absence of external reagents, is still the most studied in vivo [28,29]. Nevertheless, ferritin’s MRI performance is severely limited by its weak magnetic properties and highly conservative structure. The latter excludes significant improvement in ferritin relaxivity by bioengineering. Another promising approach is based on the heterologous expression of bacterial protein nanocompartments—encapsulins—capable of forming iron-containing nanoparticles of a size limited by the nanoshell diameter [30]. They can sequester up to 30,000 Fe atoms, which is about 10 times the capacity of ferritin. Encapsulins represent a multicomponent system with a protein that self-assembles into an icosahedral shell and cargo protein(s) targeted to the inner surface of this nanocompartment via a short encapsulation signal. In nature, encapsulins are expressed in prokaryotes and serve as nanoreactors compartmentalizing various chemical reactions [31,32]. Recently, however, it has been shown that genes encoding encapsulins can be expressed in mammalian cells. The assembly of nanocompartments with endogenous (ferroxidase) and nonendogenous cargos, such as tyrosinase and various fluorescent proteins, remains effective [33,34]. This phenomenon has been described in HEK293T cells and in vivo in mouse and *Drosophila* neurons [33,34]; expression in a dedicated tumor cell line has not been shown to date.

In this paper, we demonstrate the expression of encapsulin reporter genes from *Quasibacillus thermotolerans* in human cancer cells as a two-component system representing a nanoshell (QtEnc^FLAG^) and a cargo protein. We used either the natural ferroxidase cargo from *Q. thermotolerans* (QtIMEF), which induces iron oxide biomineralization within the nanocompartment, or a synthetic fluorescent cargo protein derived from mScarlet-I (DD-mScarlet-I-QtSig). Coexpression of QtEnc^FLAG^ and QtIMEF resulted in enhanced T_2_-weighted contrast in MRI. Also, coexpression of QtEnc^FLAG^ and DD-mScarlet-I-QtSig can provide additional fluorescent contrast for subsequent histological analysis in preclinical tumor models. In particular, we chose the human hepatocellular carcinoma cell line HepG2, which is widely used in therapeutic cancer research and drug screening [35,36]. Thus, our in vitro experiments could potentially be translated into further in vivo cancer-cell tracking.

## 2. Materials and Methods

### 2.1. Genetic Constructs

Mammalian codon-optimized QtEnc (UniProt: A0A0F5HPP7_9BACI), QtIMEF (UniProt: A0A0F5HNH9_9BACI), MmZip14^FLAG^ (Zip14, UniProt: Q75N73), and mScarlet-I (GenBank: APD76536.1) were custom-synthesized by Integrated DNA Technologies and cloned into pcDNA3.1 (+) Zeocin (Invitrogen) using restriction cloning. A FLAG epitope tag was C-terminally appended to QtEnc using Q5 site-directed mutagenesis (New England Biolabs, Frankfurt am Main, Germany) [33]. Multigene expression of QtIMEF and QtEnc^FLAG^ was achieved by generating a single reading frame containing the two genes separated by a P2A peptide, yielding QtIMEF_P2A_QtEnc^FLAG^. For targeting the encapsulin nanocompartments, mScarlet-I was C-terminally fused to a 2× GGGGS linker, followed by the minimal encapsulation signal KGFTVGSLIQ (QtSig). For generating the destabilized version of mScarlet-I, the HA-Tag (YPYDVPDYA) followed by a GT linker and L106P mutant of FKBP12 (DD-N) [34,37] followed by the GSG linker were N-terminally appended to mScarlet-I-QtSig using Gibson Assembly, yielding DD-mScarlet-I-QtSig.

### 2.2. Cell Culture and Protein Expression

Low-passage-number HepG2 (ECACC: 85011430) cells were cultured in DMEM/F-12 with 10% FBS and penicillin–streptomycin at 100 μg/mL at 37 °C and 5% CO_2_. Cells were transfected with Lipofectamine 3000 Transfection Reagent (Invitrogen) according to the manufacturer’s protocol. To express the combination of QtIMEF_P2A_QtEnc^FLAG^ and Zip14, 95% of the total DNA amount encoded the QtIMEF_P2A_QtEnc^FLAG^, and 5% of the total DNA amount encoded Zip14. To express the combination of QtEnc^FLAG^ and DD-mScarlet-I-QtSig, 65% of the total DNA amount encoded the shell, and the remaining 35% coded the fluorescent cargo. Cells were supplemented with medium containing ferrous ammonium sulfate (FAS, Sigma-Aldrich, Darmstadt, Germany) at the indicated concentrations 24 h after transfection to facilitate iron loading. For protein-expression analysis, cells were harvested 48 h after transfection and lysed with mammalian protein extraction reagent M-PER (Pierce Biotechnology, Waltham, MA, USA) containing a mammalian protease inhibitor cocktail (SIGMA P8340, Sigma-Aldrich, Darmstadt, Germany) according to the manufacturer’s protocol. After spinning down the cell debris at 14,000× *g* for 15 min, the cell lysates were incubated at 4 °C for subsequent analyses. Protein concentrations in the lysates were determined by OD measurement at 280 nm.

### 2.3. Optical Microscopy

The RFP channel (531/40 nm excitation; 593/40 nm emission) of an EVOS FL Auto microscope (Life Technologies, Carlsbad, CA, USA) with PlanFluor 20×/0.45 and 40×/0.65 objectives was used to image mScarlet-I fluorescence in HepG2 cells. To determine QtEnc^FLAG^ transfection and assembly efficiency, cells were manually counted in fluorescent and phase-contrast images. The number of fluorescent cells (expressing assembled QtEnc^FLAG^ nanoshells and DD-mScarlet-I-QtSig cargo) was divided by the total number of cells.

### 2.4. Transmission Electron Microscopy (TEM)

HepG2 cells were fixed in 2.5% EM grade glutaraldehyde in 0.1 M sodium cacodylate buffer, pH 7.4 (Science Services, Munich, Germany), and postfixed in 1% aqueous osmium tetroxide [38]. The samples were then dehydrated in gradual ethanol (25–100%), embedded in epoxy resin (Merck, Darmstadt, Germany), and cured for 72 h at 60 °C. Ultrathin sections of 70 nm were collected onto 200 mesh copper grids and stained with 0.5% uranyl acetate and 3% lead citrate before performing transmission electron microscopy (Zeiss Libra 120 Plus, Carl Zeiss NTS GmbH, Oberkochen, Germany). Pictures were acquired using a slow-scan CCD-camera and iTEM software (Olympus Soft Imaging Solutions, Münster, Germany). The average diameter of nanoparticles (NPs) was calculated from images by analysis of 100 NPs using ImageJ software.

### 2.5. Blue Native Polyacrylamide Gel Electrophoresis (PAGE)

For the detection of native encapsulins, precast NativePAGE Novex 3–12% Bis-Tris gels (Life Technologies, Carlsbad, CA, USA) were used according to the manufacturer’s protocol. Gels were loaded with whole-cell lysates mixed with NativePAGE Novex sample buffer and ran for 120 min at 150 V. Unstained protein standard (Life Technologies), covering a size range between 20 and 1200 kDa, was used as a marker. The total protein contents of whole-cell lysates loaded per well were adjusted to ∼1–3 μg. Gels were Coomassie-stained using Bio-Safe Coomassie Stain (Bio-Rad Laboratories).

### 2.6. Cell-Viability Assay

Gene- and iron-related cytotoxicity was monitored via the LDH-Glo™ Cytotoxicity Assay (Promega) according to the manufacturer’s protocol. Briefly, 5 × 10^4^ HepG2 cells were seeded on a 96-well plate. Then, 24 h post-seeding, cells were transfected with different combinations of genes using Lipofectamine 3000 Transfection Reagent (Invitrogen, Carlsbad, CA, USA). To express the combination of QtIMEF_P2A_QtEnc^FLAG^ and Zip14, 95% of the total DNA amount encoded the QtIMEF_P2A_QtEnc^FLAG^, and 5% of the total DNA amount encoded Zip14. To express the combination of QtEnc^FLAG^ and DD-mScarlet-I-QtSig, 65% of the total DNA amount encoded the shell, and the remaining 35% coded the fluorescent cargo. Next, 24 h post-transfection, FAS was added to the medium with the cells coexpressing QtIMEF_P2A_QtEnc^FLAG^ and Zip14 from a 100 mM FAS stock solution, yielding final concentrations in the 0–2 mM range. Then, 24 h after addition of FAS, cells were assayed for LDH release. The cell-viability assay was performed in a 96-well plate format as an endpoint measurement. Luminescence readings were taken on a Centro LB 960 (Berthold Technologies, Bad Wildbad, Germany) at 0.5 s acquisition time. The viability of untransfected cells treated with 0.2% Triton X-100 was taken as 0%.

### 2.7. Magnetic Resonance Imaging of Cells

MR images were acquired with a Bruker BioSpec 94/20USR, 9.4T system equipped with an RF RES 400 1H 112/072 Quad TR AD resonator. For T_2_ and T_2_* measurements of cell pellets, 5 × 10^5^ HepG2 cells were seeded 24 h before transfection on a 6-well plate. Then, 24 h post-transfection, FAS was added to the medium, yielding a concentration of 2 mM. Next, 24 h after iron addition, cells were washed 3 times with DPBS and detached with Accutase® and centrifuged at 500× *g* for 4 min. The pellets (8 × 10^6^ cells each) were resuspended in 200 μL DPBS and transferred in 200 μL PCR tubes. Cells were then spun down at 2000× *g* for 2 min and immediately used for MRI. T_2_ and T_2_* measurements were conducted in a custom-made holder filled with DPBS to avoid susceptibility artifacts. T_2_ values were measured with a T_2_-weighted multi-slice-multi-echo (MSME) sequence with a TR of 6000 ms, 30 echoes with an echo spacing of 4.7 ms (4.7–150 ms), a flip angle of 90° for excitation and 180° for refocusing, a field-of-view (FOV) of 5 × 5 cm, and a matrix size of 256 × 256. T_2_* measurements were carried out with an ultra-short echo time (UTE) sequence with a TR of 100 ms, 16 echoes (0.3, 0.5, 1, 2, 3, 4, 5, 10, 15, 20, 25, 30, 35, 40, 45, and 50 ms), a flip angle of 15°, an FOV of 5 × 5 cm, and a matrix size of 98 × 98 pixels. Relaxation rates were calculated by fitting a monoexponential function to the measured sample intensity values using the Image Sequence Analysis Tool, ParaVision 6.0.1 (Bruker BioSpin MRI GmbH, Ettlingen, Germany).

### 2.8. Inductively Coupled Plasma Mass Spectrometry (ICP-MS)

Measurements were performed on a NexION 350D (PerkinElmer, Waltham, MA, USA) in collision mode with kinetic energy discrimination. HepG2 cell pellets previously used for the MRI measurements (8 × 10^6^ cells each) were dissolved in 50 μL of 65% nitric acid for 2 h at 65 °C, then incubated at room temperature overnight and afterward diluted 1:10 with deionized water.

## 3. Results

We investigated the heterologous expression of encapsulin reporter genes from *Quasibacillus thermotolerans* in human cancer cells, starting with the coexpression of encapsulin nanocompartments with the fluorescent reporter cargo protein. For this purpose, HepG2 cells were transiently cotransfected with the DNA of QtEnc^FLAG^ nanoshell and DD-mScarlet-I-QtSig fluorescent cargo. The DD-mScarlet-I-QtSig construct was used to approximate the quantification of assembled QtEnc^FLAG^ because the destabilizing domain labels the mScarlet-I cargo for rapid proteasomal degradation if it is not shielded through encapsulation into the QtEnc^FLAG^ nanocompartment [34].

**Figure 1:**
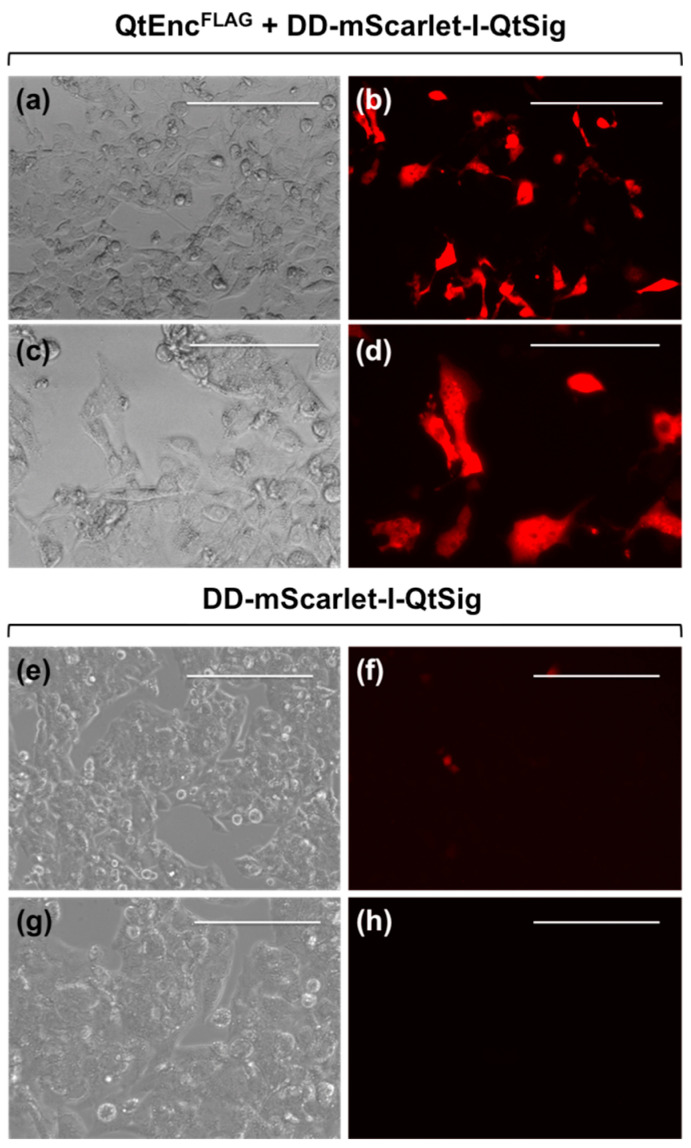
Fluorescent microscopy images of HepG2 cells expressing QtEnc^FLAG^ nanoshell and DD-mScarlet-I-QtSig (QtEnc^FLAG^ + DD-mScarlet-I-QtSig) cargo protein (**a**–**d**) and DD-mScarlet-I-QtSig cargo protein alone (**e**–**h**). (**a**,**c**,**e**,**g**): Differential interference contrast microscopy images; (**b**,**d**,**f**,**h**): RFP-channel fluorescence images. The scale bars in (**a**,**b**,**e**,**f**) correspond to 200 μm; the scale bars in (**c**,**d**,**g**,**h**) correspond to 100 μm.

Indeed, we detected a strong fluorescent signal in the RFP channel for ≈20% of HepG2 cells coexpressing QtEncFLAG and DD-mScarlet-I-QtSig (Figure 1a–d). HepG2 cells expressing only DD-mScarlet-I-QtSig gave a weak background signal (Figure 1e–h). Thus, about 20% of cells express correctly assembled QtEnc^FLAG^ nanocompartments with mScarlet-I protein fluorescence sequestered into the encapsulin shell.

To evaluate iron biomineralization within encapsulins expressed in HepG2 cells, we cotransfected QtIMEF_P2A_QtEnc^FLAG^, encoding QtEnc^FLAG^ nanoshell and QtIMEF cargo with the ferroxidase activity and the iron transporter Zip14. After 24 h, the cells were supplemented with 2 mM FAS for another 24 h and then prepared for TEM.

**Figure 2:**
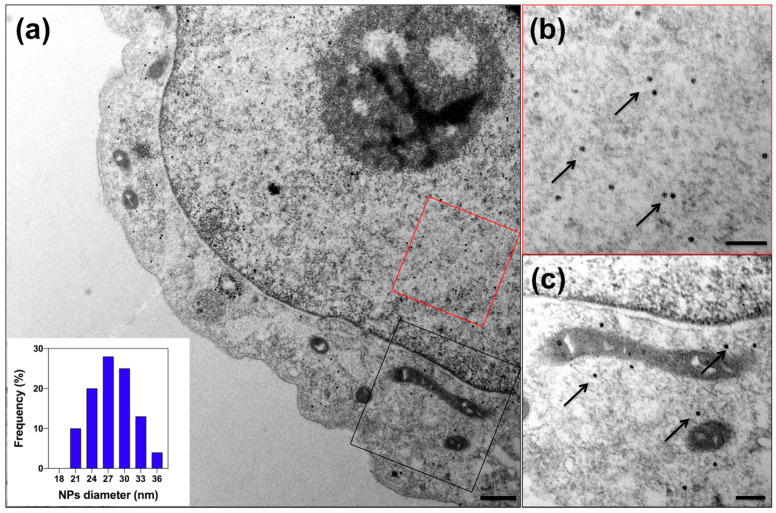
TEM images of the HepG2 cells transiently expressing QtEnc^FLAG^ with QtIMEF cargo protein (QtIMEF_P2A_QtEnc^FLAG^) and iron transporter Zip 14 cultivated in the medium supplemented with 2 mM ferrous ammonium sulfate (FAS) for 24 h. (**a**) Overview TEM image; the scale bar represents 500 nm. Close-up of exemplary regions in the nucleus ((**b**), red rectangle) and cytosol ((**c**), black rectangle)) with arrows pointing to the individual nanoparticles (NPs) containing iron; the scale bar represents 200 nm. The inset in (**a**) illustrates the size distribution of iron oxide cores inside the encapsulin shells.

Microscopic images of ultrathin sections showed intact cells—notably, the integrity of membranes and mitochondria, medium density-cytoplasm with ribosome inclusions, and absence of large vacuoles (Figure 2). Also, the cells contained electron-dense iron oxide nanoparticles due to FAS oxidation and Fe sequestration within the nanoshell. This finding is similar to the data presented for HEK293T cells by Sigmund et al. [33], although with a significantly lower expression level. Iron oxide cores had a narrow size distribution with an average diameter of 27.7 ± 3.9 nm. Interestingly, QtEnc^FLAG^ nanoshells could be detected in the cell cytoplasm and the nucleoplasm, with iron biomineralization occurring in both cases.

In addition, the expression and assembly of QtEnc^FLAG^ nanoshells were demonstrated biochemically in HepG2 cell lysates. Coomassie-stained Blue Native PAGE (Figure S1) revealed a band with an apparent molecular weight above 1.2 MDa corresponding to T = 4 assembly of QtEnc^FLAG^ with the same electrophoretic mobility in the case of encapsulated cargo proteins QtIMEF and DD-mScarlet-I-QtSig. In contrast, lysates of nontransfected cells did not contain a band with this molecular weight. Compared to HEK293T cells [33], the bands’ intensities were significantly less pronounced, indicating the lower expression levels of encapsulin proteins.

To investigate the possible cytotoxic effects of Fe loading in encapsulins, we performed an LDH release assay. Cells were cotransfected with QtIMEF_P2A_QtEnc^FLAG^ and Zip14 and cultured in a medium supplemented with FAS at a concentration range of 0 to 2.0 mM for 24 h, or cotransfected with QtEnc^FLAG^ and DD-mScarlet-I-QtSig and cultured in medium without Fe supplementation for 24 h (Figure S2). Coexpression of QtEnc^FLAG^, DD-mScarlet-I-QtSig, and Zip14 was not tested due to the high probability of oxidative stress caused by intense FAS consumption in cells enhanced by Zip14 without QtIMEF, allowing biomineralization of iron oxide within QtEnc^FLAG^ nanoshells.

Transfection itself reduced cell viability to 94.4 ± 1.5% for cells coexpressing QtIMEF_P2A_QtEnc^FLAG^ and Zip14, and 93.5 ± 1.3% for cells coexpressing QtEnc^FLAG^ and DD-mScarlet-I-QtSig. In both cases, cells were cultured in DMEM with 0 mM FAS. The effect of iron supplementation led to a slight dose-dependent decrease in cell viability to 90.0% for cells coexpressing QtIMEF_P2A_QtEnc^FLAG^ and Zip14 cultured in DMEM with 2 mM FAS.

**Figure 3:**
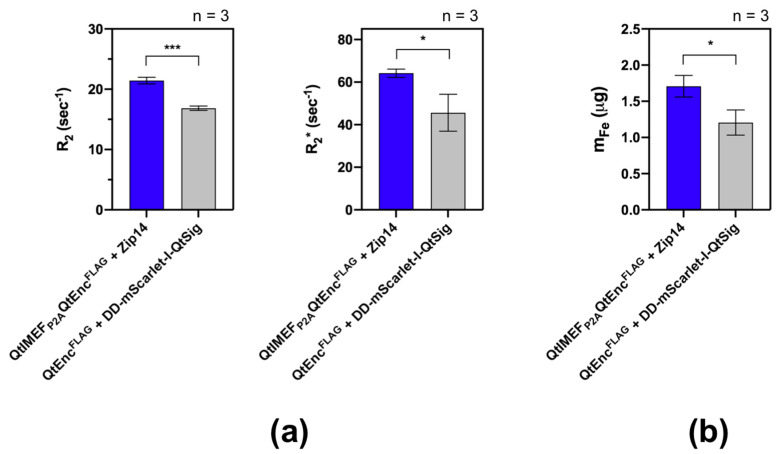
MRI relaxometry of the HepG2 cells transiently expressing the constructs as indicated in the figure and cultivated in the medium supplemented with 2 mM FAS for 24 h. (**a**) R_2_ and R_2_* values were computed from cell pellets comprising ~8 × 10^6^ cells. (**b**) Fe masses in the same cell pellets determined by inductively coupled plasma mass spectrometry (ICP-MS). All numbers are plotted as mean values ± SD (*n* = 3). Statistical analysis was performed by unpaired *t*-test (*** corresponds to *p*-value < 0.001, * corresponds to *p*-value < 0.05).

Next, we were interested in whether iron accumulation within the encapsulins would significantly increase the cancer cells’ contrast in MRI. As the first step in this direction, we performed relaxometry measurements of HepG2 cell pellets at 9.4 T (Figure 3). In the test sample, cells were transfected with QtIMEF_P2A_QtEnc^FLAG^ and Zip14; as a control sample, we used HepG2 cells expressing QtEnc^FLAG^ and DD-mScarlet-I-QtSig because fluorescent cargo does not result in Fe biomineralization within the nanoshell. In both cases, cells were cultured in a medium supplemented with 2 mM FAS for 24 h.

A statistically significant increase in R_2_ and R_2_* (Figure 3a) of the test sample with QtIMEF cargo compared to the control sample with DD-mScarlet-I-QtSig cargo was found (R_2_ = 21.4 ± 0.6 vs. 16.8 ± 0.4 s^−1^ and R_2_* = 64.2 ± 2.0 vs. 45.6 ± 8.6 s^−1^). This finding was consistent with the difference in Fe accumulation between these cell samples tested by ICP-MS (Figure 3b), corresponding to additional Fe biomineralization in encapsulin nanoshells.

## 4. Discussion

In the present study, we investigated the expression and assembly of encapsulin nanocompartments from *Q. thermotolerans* and their properties for packaging various cargo proteins. The latter, in particular, led to iron biomineralization and derived MR effects in the mammalian tumor cell line HepG2. As a fluorescent cargo protein, we chose mScarlet-I [39,40], targeted to the QtEnc^FLAG^ nanoshell via QtSig encapsulation signal and modified by the DD domain (DD-mScarlet-I-QtSig). The latter ensures mScarlet-I fluorescence only within the encapsulin nanocompartment, while the fluorescent signal of transfected cells remains relatively high. These data suggest that non-natural cargo can be efficiently targeted to the encapsulin nanocompartments in mammalian cancer cells. With this targeting of fluorescent molecules, it is possible to assess the expression efficiency of the encapsulin nanocompartments and isolate cells with the most robust expression using fluorescence-activated cell sorting (FACS). Such reporter proteins are contrast-selective for viable cells with normal metabolism and are not diluted by cell division. These advantages make genetically encoded fluorescent probes for cancer cell labeling and detection (in particular, HepG2 cells) more efficient compared to synthetic fluorophores and aptamer-based probes [41,42], as well as quantum dots [7].

Next, we focused on iron biomineralization by the natural cargo protein QtIMEF with ferroxidase activity enhanced by coexpression of the iron transporter Zip14. Indeed, TEM images proved the formation of iron oxide nanoparticles with high density, spherical shape, and monodisperse distribution within QtEnc^FLAG^ nanoshells. We confirmed the self-assembly of QtEnc^FLAG^ nanocompartments with QtIMEF or DD-mScarlet-I-QtSig cargo proteins after transient transfection by Blue Native PAGE analysis. We demonstrated high viability of cells coexpressing either QtIMEF_P2A_QtEnc^FLAG^ and Zip14 in medium supplemented with 0–2 mM FAS or coexpressing QtEnc^FLAG^ and DD-mScarlet-I-QtSig in standard DMEM medium. In all cases, viability did not fall below 90%. The TEM data, on-gel analysis of encapsulin expression, and viability results were consistent with previously reported data on HEK293T cells [33,34].

Finally, in MRI relaxometry measurements, we demonstrated increased R_2_ and R_2_* relaxation rates for cells coexpressing QtIMEF_P2A_QtEnc^FLAG^ and Zip14, consistent with higher Fe accumulation by these cells as confirmed by ICP-MS. Despite the lower levels of encapsulin protein expression in HepG2 cells, the data obtained replicated similar findings in the HEK293T cell line [34]. It should be noted that the relaxation rates of encapsulin nanocompartments are lower than those of synthetic magnetic nanoparticles and magnetoliposomes of a similar size, representing ideal magnetite in vitro [43,44]. Interestingly, our R_2_ values are of the same order of magnitude as the R_2_ of nanoparticles derived from magnetotactic bacteria at the same concentration and used for MRI-guided photothermal therapy of HepG2 tumor [45]. Our approach does not require the addition of exogenous magnetic particles, since the encapsulin system is encoded in tumor cells that transmit reporter genes to the next generations of cells, which is a distinct advantage. The efficiency of encapsulin protein expression in HepG2 cells should be further improved, e.g., by using viral transduction and generating a stable cell line. Within the latter, it is conceivable to use the selection of cells with the most pronounced encapsulin expression via magnetic-activated cell sorting (MACS), similar to the method described in [34], which may then enable in vivo cell tracking by MRI.

To conclude, we have shown the heterologous expression of the encapsulin system from *Q. thermotolerans* with the fluorescent reporter protein mScarlet-I and the iron-mineralizing protein IMEF in the human hepatocellular carcinoma cell line HepG2. We highlighted results from fluorescence microscopy, TEM, native gel electrophoresis, cell-viability assay, and T_2_-relaxivity studies. There is potential for further improvements in this genetically encoded approach to enable in vivo cell tracking in cancer models by MRI and optical techniques.

## Data Availability

The data presented in this study are available on request from the corresponding author.

## Supplementary Materials

The following are available online at https://www.mdpi.com/1999-4923/13/3/397/s1, Figure S1: Expression of encapsulins in cancer cells, Figure S2: The results of the LDH assay.
