# Supplementary material for: Genetically Encoded Self-Assembling Iron Oxide Nanoparticles as a Possible Platform for Cancer-Cell Tracking
Source: Pharmaceutics. 2021 Mar 16;13(3):397. doi: 10.3390/pharmaceutics13030397 (PMC8002387; doi:10.3390/pharmaceutics13030397)
Supplement: Supplementary file 1 [file pharmaceutics-13-00397-s001.pdf]

pharmaceutics

Supplementary Materials: Genetically Encoded Self-Assembling Iron Oxide Nanoparticles as a Possible Platform for Cancer-Cell Tracking

Maria V. Efremova, Silviu-Vasile Bodea, Felix Sigmund, Alevtina Semkina, Gil G. Westmeyer and Maxim A. Abakumov

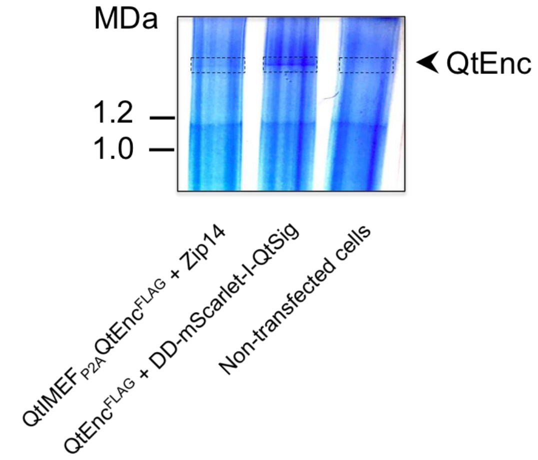

Figure S1. Coomassie-stained Blue Native PAGE gels loaded with cell lysates of HepG2 cells expressing QtIMEF$_{P2A}$QtEnc^{FLAG} + Zip14 or QtEnc^{FLAG} + DD-mScarlet-I-QtSig, and the lysate of non-transfected cells. All cells were supplemented with 2 mM ferrous ammonium sulfate (FAS) for 24 h. Arrows and frames indicate the bands corresponding to the assembled QtEnc nanoshells that are absent in the lysate of non-transfected cells.

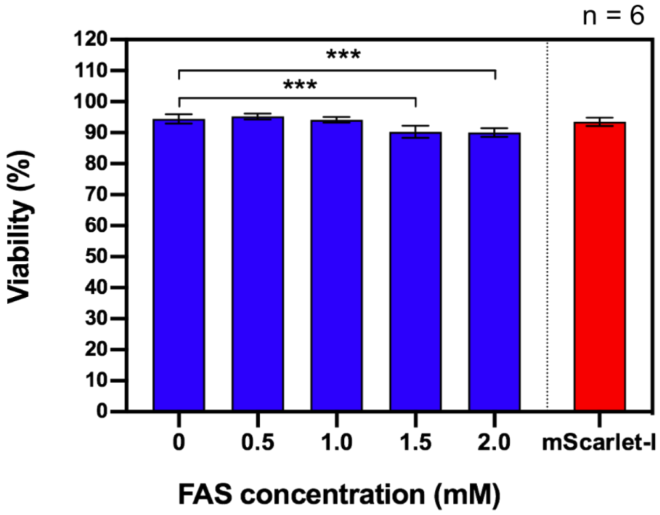

Figure S2. The results of the LDH assay. HepG2 cells were either co-transfected with QtIMEF$_{P2A}$QtEnc^{FLAG} and Zip14 and cultured in the medium supplemented with FAS in 0–2.0 mM concentration range for 24 h (blue columns) or co-transfected with QtEnc^{FLAG} and DD-mScarlet-I-QtSig and cultured in the medium without Fe supplementation for 24 h (red column). The viability of untransfected cells treated with 0.2% Triton X-100 was taken as 0%. The numbers are plotted as mean values ± SD (n = 6 for each condition). Statistical analysis was done by ordinary one-way ANOVA with Bonferroni's multiple comparisons test (*** corresponds to p-value < 0.001).

Publisher's Note: MDPI stays neutral with regard to jurisdictional claims in published maps and institutional affiliations.

Copyright: © 2021 by the authors. Submitted for possible open access publication under the terms and conditions of the Creative Commons Attribution (CC BY) license (http://creativecommons.org/licenses/by/4.0/).
